# Phage treatment significantly reduces *Salmonella* shedding in layer hens and *Salmonella* abundance on the surface of the eggs they produce

**DOI:** 10.1016/j.psj.2026.106416

**Published:** 2026-01-09

**Authors:** Anisha M. Thanki, Natasha Whenham, Tom Dale, Mike R. Bedford, Helen V. Masey O’Neill, Martha R.J. Clokie

**Affiliations:** a Becky Mayer Centre for Phage Research, Division of Microbiology and Infection, University of Leicester, Leicester LE1 7RH, UK; b AB Agri, Innovation Way, Peterborough Business Park, Peterborough PE2 6FL, UK; c AB Vista, Woodstock Court, Marlborough Business Park, Marlborough, Wiltshire SN8 4AN, UK

**Keywords:** Poultry, Bacteriophages, Phage therapy, *Salmonella*, Novel antimicrobials

## Abstract

Non-typhoidal *Salmonella* species cause 85% of human foodborne cases and infections caused by multidrug-resistant *Salmonella* strains are increasing. Therefore, alternative antimicrobials are needed, and phage therapy offers a promising tool to reduce the spread of *Salmonella* in the human food chain. The aim of this study was to determine if a phage cocktail delivered in water could reduce *Salmonella* shedding in infected egg laying hens, and *Salmonella* abundance on the surface of eggshells in eggs produced by infected hens. 240 56-week-old layer hens, which were environmentally infected with *Salmonella* were divided into three treatment groups: T1, no phage cocktail, T2, phage cocktail at dose 3×10^6^ PFU/day and T3, phage cocktail at dose 3×10^5^ PFU/day. The phage cocktail was delivered in their drinking water, for 28 days. Our study found by day 28 the higher phage dose (T2) was more efficient at reducing *Salmonella* shedding (p<0.01) and median *Salmonella* shedding counts were 3.10×10^4^ and 0.00 CFU/g for groups T1 and T2 respectively. In the eggs produced by T2 phage treated hens, *Salmonella* abundance on eggshells was reduced by 60% (p<0.01) compared to the eggs produced by infected hens that received no phage treatment. We showed phage treatment effectively reduced *Salmonella* transmission in laying hens and in their eggs. Our data highlights that phage treatment is a promising tool to improve food safety.

## Introduction

Globally, each year, nontyphoidal *Salmonella* spp are responsible for 93 million cases of gastroenteritis and 155,000 deaths of which 85% relate to consumption of contaminated food (Lamichhane et al*.,* 2024). Over 20% of these infections are linked to *Salmonella* contaminated pork, poultry meat and products, including eggs. The worldwide related health and economic burden is estimated at ∼$3 billion annually (Solís et al*.,* 2023). Therefore, to protect public health, it is imperative to control the spread of *Salmonella* in the food chain.

Over 2500 *Salmonella* serotypes have been identified but not all are linked to poultry and human enteric infections. Prevalent infecting serotypes differ based on time and geographical location (Chen et al*.,* 2024). Currently, *S. enterica* serovars *S*. Typhimurium, *S.* Enteritidis**,** *S*. Idikan, *S*. Infantis, *S.* Kedougou, *S.* Heidelberg and *S.* Kentucky are dominant and have been linked to poultry outbreaks and subsequent human infections across the world (Chard et al*.,* 2025; Shah et al., 2017).

During the poultry production cycle chickens and laying hens can become infected with *Salmonella* through consumption of contaminated feed, water or litter, or through aerosol transmission in pens and by contact with *Salmonella* infected carrier animals such as insects and rodents (Terio et al*.,* 2023). Furthermore, laying hens' genetics, diet, environment and intestinal microbiome are significant factors that predispose hens to *Salmonella* infection. Eggs produced by infected layer hens can become contaminated with *Salmonella* through the transovarial route where the infection originates in the reproductive organs and occurs in the yolk before the egg is laid (Gast et al*.,* 2024). Eggs can also become contaminated via the faecal-oral route in where *Salmonella* cells contaminate eggshells as they pass through the hens colonised gastrointestinal tract (Shaji et al*.,* 2023).

In the United Kingdom (UK) poultry eggs and egg products were implicated in 26% of all *Salmonella* foodborne outbreaks (18/68) between 2015-2020 and in the European Union (EU) in 2021 were responsible for 44% (37/84) of outbreaks (FDA, 2023). Antibiotics penicillin, tetracycline and chloramphenicol are routinely used to control and treat *Salmonella* infections (Solís et al*.,* 2023). However, in many places such as the US, EU and the UK the use of antibiotics as growth promoters have been banned and there are government incentives to curtail antibiotic use in agriculture altogether (Castanon, 2007). This is to reduce the spread and burden of antibiotic resistance in the food chain, which subsequently impacts human health (Rahman and Hollis, 2023). In addition, antibiotics are not bacterial species specific and can disrupt the poultry microbiome leading to further enteric infections (Liang et al*.,* 2023). Therefore, alternative control strategies in addition to increased farm biosecurity are being considered to prevent the spread of *Salmonella* in poultry production. These include vaccination of birds and prebiotics, probiotics, synbiotics and postbiotics (Shaji et al*.,* 2023). However, with all strategies, efficacy data to reduce *Salmonella* has been inconsistent, thus further research is needed before they can be widely implemented by the industry (Abd El-Hack et al*.,* 2022).

Bacterial viruses, bacteriophages (phages), could also provide a promising alternative preventative or treatment to antibiotics. Phages are bacterial species specific and therefore only lyse the target bacteria without disrupting the remaining microbiome. They are also harmless to human and animal cells. Lytic phages lyse their target bacterial host by taking over the host’s machinery to replicate which leads to bacterial cell death and the release of further infecting phages (Clokie et al*.,* 2011). Lytic phages can be used therapeutically against human and animal bacterial pathogens and collective therapeutic data has highlighted their effectiveness, efficacy and safety (Węgrzyn, 2023; Cui et al*.,* 2024).

*Salmonell*a challenge poultry studies have highlighted that phage or phages combined as cocktails delivered orally, in feed or in water are effective at reducing *Salmonella* infection in infected broiler birds (Thanki et al*.,* 2021; Gigante and Atterbury, 2019). For example, a study showed birds experimentally infected with *Salmonella* Enteritidis and treated with phage cocktail ProBe-Bac® in feed available *ad libitum* for 28 days reduced *Salmonella* colonisation by 1.5 log_10_ CFU/ml between 7-14 days post-infection (n=30). The authors tested two phage doses with inclusion in the basal diet at 1 g/kg and 1.5 g/kg (phage titre was not stated), and the latter inclusion rate was most effective in reducing *Salmonella* abundance in their caeca and liver (p<0.05) (Sarrami et al*.,* 2023). Phages are also effective at reducing *Salmonella* abundance in eggs. Braz et al (2025) showed applying a single phage phSE-5 at dose 10^8^ PFU/mL in liquid whole eggs reduced *S*. Typhimurium abundance by 1.8 log_10_ CFU/mL after 12 h. Furthermore, when the phage was sprayed on eggshells at the same dose it caused a 1.3 log_10_ CFU/mL reduction in *Salmonella* counts after 8 hours (Braz et al*.,* 2025). Although these and published studies show phages are efficacious against *Salmonella* the optimal phage dose is unknown and likely could be phage specific.

We have developed a two-phage cocktail, which includes phages SPFM10 and SPFM14 part of the Seulviruses genus against *Salmonella*. Our phage cocktail can effectively lyse multiple serotypes of *Salmonella* associated with infections in poultry and swine, which are also responsible for human food poisoning (Thanki et al*.,* 2019; Thanki, Clavijo et al*.,* 2022; Thanki et al*.,* 2022). This includes the prevalent poultry infecting serotypes *S*. Typhimurium, monophasic *S*. Typhimurium, *S*. Enteritidis, *S*. Idikan, *S*. Kedougou, *S*. Derby and *S*. Bovismorbificans (Thanki et al., 2021). Furthermore, we showed when the phage cocktail was delivered in feed to experimentally challenged broiler birds, phage treatment significantly reduced *Salmonella* shedding in infected birds (p<0.05). Similar to the poultry study discussed above, optimal dose was unknown and we tested three doses of the phage cocktail at 10^5^ PFU/day, 10^6^ PFU/day and 10^7^ PFU/day in feed, which were all available *ad libitum* for 42 days. We found the lowest dose 10^5^ PFU/day was the most effective and reduced *S.* Typhimurium to below detectable limits levels in all birds by day 42. The higher doses also significantly reduced *Salmonella* shedding (p<0.05) (Thanki et al*.,* 2023).

These experimental poultry challenge studies demonstrate the effectiveness of phage therapy (Melaku et al*.,* 2025). However, there is limited data on phage efficacy to treat *Salmonella* infected layer hens and if treating layer hens with phages can also reduce *Salmonella* abundance on the surface of eggs they produce. The aim of this study was to determine if the phage cocktail SPFM10-SPFM14 we have developed can reduce *Salmonella* colonisation and shedding in layer hens on a commercial farm, and if it reduces the *Salmonella* burden associated with the eggs they produce.

Unlike most previous studies that have evaluated phage efficacy using experimentally challenged birds or artificially inoculated eggs, our work investigated a naturally infected population of layer hens maintained under typical farm conditions. This setting, common in many parts of the world, provided a realistic test of phage performance under environmental infection pressures rather than controlled laboratory challenge. Despite the unknown serotype(s) of infecting *Salmonella* and the absence of prior host–phage matching, we wanted to determine if our cocktail could reduce *Salmonella* colonisation and shedding when administered via drinking water at 10⁶ PFU/L and 10^5^ PFU/day. Our study highlights the higher dose was most effective at reducing *Salmonella* abundance in infected layer hens and in the eggs produced by these hens *Salmonella* counts on the surface of the eggshell were significantly reduced.

## Methods and materials

### *Salmonella* strain and phages used in this study

The *Salmonella enterica* subsp. enterica serovar Typhimurium SL1344 strain was used as the phage propagating strain. The strain was routinely grown on Xylose Lysine Deoxycholate (XLD) agar (Oxoid, UK) medium at 37°C overnight on which *Salmonella* produces distinct black colonies. Liquid cultures of SL1344 were made by inoculating single colonies grown on XLD media into Luria broth (LB) (Melford, UK) and cultures were incubated overnight at 37°C whilst being shaken at 100 rpm. Phages SPFM10 and SPFM14, are both part of an international Leicester patent ‘Therapeutic Bacteriophages’ and were used for this study (PCT/GB2019/052695).

### Phage propagation and titration

Phages SPFM10 and SPFM14 were propagated individually in LB broth as previously described (Thanki et al*.,* 2023). Briefly, SL1344 liquid cultures were diluted 1/100 in 1 L of LB broth and grown to optical density 0.2 (wavelength 600 nm). After which 10 mL of phage at titre ∼10^8^ plaque forming units per mL (PFU/mL) was added. Phage and bacterial cultures were grown for 6 hours at 37°C at 100 rpm, centrifuged at 4200 x *g* for 15 minutes and filtered with 0.2 micron pore size filters. Phage lysates were diluted 10-fold in SM buffer (100 mM NaCl, 8 mM MgSO_4_·7H_2_O, and 50 mM Tris-Cl) to calculate phage titres. Dilutions were plated using the plaque assay method on LB 1% (w/v) agar plates on a lawn of SL1344 and plates were incubated at 37°C overnight (Kutter, 2009). A total volume of 12 L of phages were produced, and each phage was at a titre of 3×10^8^ PFU/mL.

### Layer hen trial

The laying hen trial was conducted at Poultry Research Farm Ltd in India in accordance with the animal welfare practices in India and approved by the Committee for Control and Supervision of Experiments on Animals.

At the start of the study layer hens were 56 weeks of age, which will be referred to as day 0 of the study. The study was conducted for 4 weeks at which point hens were 60 weeks of age and will be referred to as day 28 of the study. In this study a total of 240 female Hyline layer hens were used and were equally divided into three treatment (T) groups i.e. 80 hens per treatment group. Hens were housed in open sided poultry houses in cages. Four hens were housed per cage and there were 20 cages per treatment group. Feed (Table 1) and water was available *ad libitum* throughout the study. Layer hens were not challenged with *Salmonella* and were already infected, confirmed by the poultry site. The groups were: T1 *Salmonella* infected, no phage; T2 *Salmonella* infected, phage treatment at a dose of 10^6^ PFU/day; T3 *Salmonella* infected, phage treatment at a dose of 10^5^ PFU/day. Phages SPFM10 and SPFM14 were mixed at equal volumes, added to drinking water and available *ad libitum* via nipple drinkers.

**Table 1:** Ingredient and nutrient composition of the feed diet fed to layer hens.

Ingredient	g/kg
Maize	533.5
De-oiled rice bran	122.34
Soybean meal	123.5
Distillery dried grain with soluble	50
Rape seed meal	40
Lime stone powder	0
Stone grit 32% (w/w)	108
Dicalcium phosphate	13.94
Common salt	3.04
Soda Bicarbonate	0.72
DL Methionine	1.16
L Lysine HCl	0.7
Trace minerals	1
Vitamin premix	0.5
Toxin Binder	1
Choline Cl	0.5
Vitamin E – Se	0.1
Nutrients	
Energy (kcal/kg)	2400
Protein %	15.5
Calcium %	3.9
aP%	0.36
% Dig Lys	0.62
% Dig Meth	0.34
% Dig M+c	0.56
% Dig thr	0.44
% Sodium	0.16

**Fig. 1:**
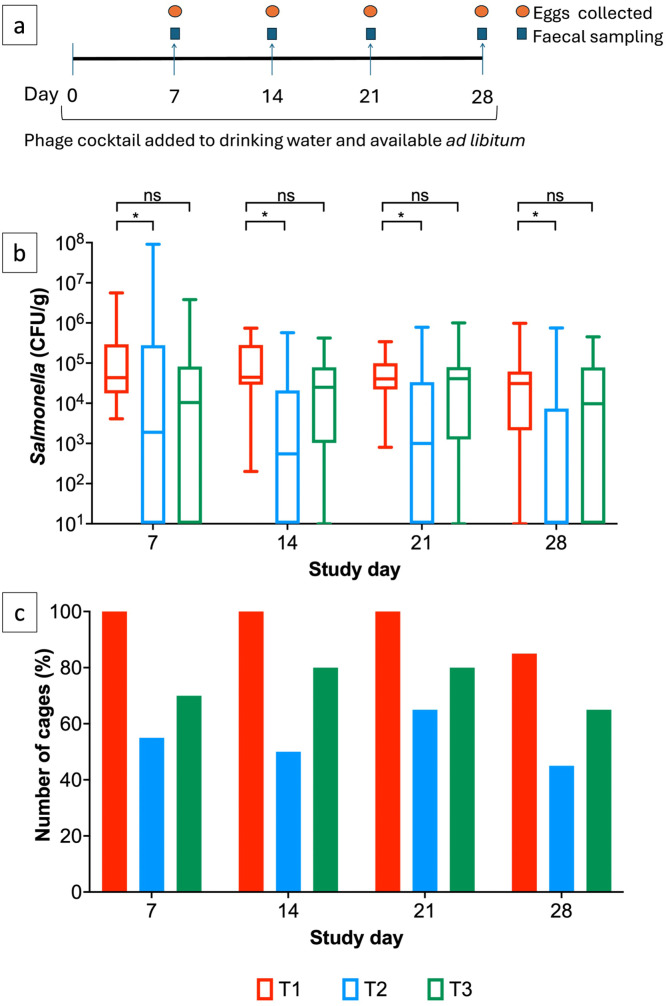
Image (a) shows the phage layer hen trial plan. *Salmonella* abundance (b) in faecal samples collected weekly over 28 days and (c) the percentage of cages positive for *Salmonella*. Statistical differences between treatment groups (n=20) are displayed on the graph (ns p>0.05 and * p<0.05).

Faecal samples were processed to monitor *Salmonella* abundance as described previously (Thanki et al*.,* 2023). Briefly, 1 g of faecal sample was weighed in sterile plastic tube, followed by the addition of 9 mL Buffered peptone water (BPW) and diluted 10-fold in BPW. Dilutions were plated on circular XLD plates, on which 0.1 mL was added and uniformly spread using L-shaped spreaders. Plates were incubated overnight at 37°C and *Salmonella* abundance was expressed as CFU/g. Faecal samples were collected on days 7, 14, 21 and 28. The study plan is shown in Fig. 1a.

To determine *Salmonella* abundance on the surface of the eggshell, each individual egg was immersed in 15 mL of BPW in a sterile plastic bag. The egg was then massaged for 2 minutes after which the eggshell rinse was collected in a sterile tube and then processed for enumeration for total *Salmonella* abundance. The wash sample was diluted 10-fold in BPW and 0.1 mL was plated on circular XLD plates. Plates were incubated overnight at 37°C and *Salmonella* abundance was expressed as CFU/egg.

### Egg wash study

Eggshell surfaces were swabbed and processed as detailed above to determine *Salmonella* abundance. Swabs were placed in 2 mL BPW, diluted 10-fold in BPW, and plated on XLD media. Plates were incubated overnight at 37°C. All *Salmonella* positive eggs were arranged in trays in the Biosafety Cabinet, the phage cocktail was sprayed at dose 10^6^ PFU/litre onto the surface of the eggs and stored for 3 hours. The phage cocktail was applied at 0.5 mL/s with a manual spray to ensure even coverage around the eggshell surface area, which was approximately 80 cm^2^/egg. After 3 hours contact time all the eggs were swabbed and placed in 2 mL BPW, then the swabs were processed for enumeration of *Salmonella* as described above.

### Statistical analysis

To determine statistical differences in *Salmonella* abundance between treatment groups one-way ANOVA was conducted and student T-tests were performed. *P* values <0.05 were noted as significant. The program Prism 9 version 9.0.2 (134) was used for analysis. Layer hen performance data and egg quality data were analysed with JMP Pro 14 and *p* values <0.05 were considered as significant.

## Results

### *Salmonella* shedding in layer hens

To determine if phage treatment could reduce *Salmonella* colonisation and shedding in infected layer hens, phage cocktail SPFM10-SPFM14 was delivered in water at two doses: 10^6^ PFU/day (T2) and 10^5^ PFU/day (T3) (Fig. 1a). Prior to study start, caecal swabs were taken from layer hens to identify which were infected with *Salmonella* and 240 *Salmonella* positive hens were included in the trial. Hens had average *Salmonella* abundance of 4×10^6^ CFU/g, though the infecting *Salmonella* serotype was unknown.

After 7 days post phage treatment the median *Salmonella* abundance in faecal samples were 4.33×10^4^, 1.90×10^3^ and 1.04×10^4^ CFU/g for T1 (*Salmonella* infected, no phage), T2 (*Salmonella* infected, phage dose 10^6^ PFU/day) and T3 (*Salmonella* infected, phage dose 10^5^ PFU/day) respectively. *Salmonella* was isolated from 100% of cages in group T1 (20/20). In comparison, *Salmonella* in the phage-treated groups T2 and T3 was reisolated in 55% (11/20) and 70% (14/20) cages respectively (Fig. 1b and c).

On days 14 and 21, average *Salmonella* abundance was significantly lower in T2 in comparison to T1 (p<0.05) and *Salmonella* was only isolated from 50-65% of cages versus 100% in T1. Median abundance counts were 4.44×10^4^ CFU/g, 5.50x10^2^ CFU/g for groups T1 and T2 respectively. In T3 samples, medium abundance was lower than T1 at 2.5×10^4^ CFU/g but not significantly different (p>0.05) and *Salmonella* was isolated from 80% (16/20) of cages (Fig. 1b and c). On the last day of the study on day 28, median *Salmonella* abundance counts were 3.10×10^4^, 0.00 (below the detection limit) and 9.75×10^3^ CFU/g for T1, T2 and T3 respectively. Counts were significantly lower in T2 (p<0.05) and *Salmonella* was reisolated from fewer numbers of cages in phage treated groups in 45% (9/20) and 65% (13/20) of cages in groups T2 and T3 (Fig. 1b and c).

### *Salmonella* abundance on eggshell surfaces

**Fig. 2:**
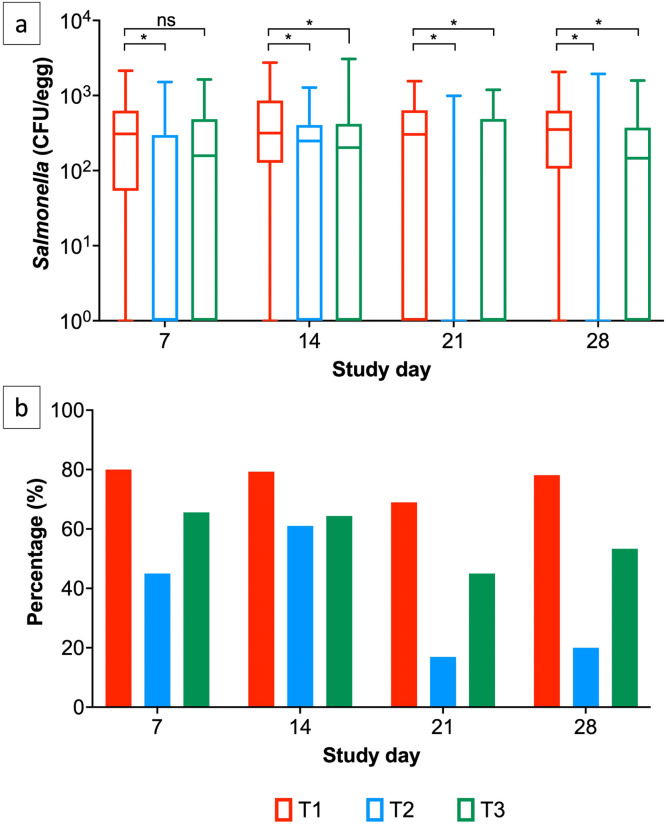
(a) *Salmonella* abundance on eggshell surface in eggs produced by T1 and phage treated layer hens (T2 and T3) and (b) percentage of *Salmonella* positive eggs. Statistical differences between treatment groups (n=60) are displayed on the graph (ns p>0.05 and * p<0.05).

Eggs produced by phage treated layer hens were screened for *Salmonella* at days 7, 14, 21 and 28 (Fig. 2). 60 eggs were screened per group on each collection day. At all sampling days after phage treatment began, *Salmonella* abundance was significantly lower in eggs produced by layer hens in T2 (*Salmonella* infected, phage dose 10^6^ PFU/day) in comparison to T1 (*Salmonella* infected, no phage). After 14 days there were also significant reductions in *Salmonella* abundance on eggshell on eggs produced by layer hens in T3 (*Salmonella* infected, phage dose 10^5^ PFU/day). Furthermore, the percentage of eggs positive for *Salmonella* were statistically consistently lower in eggs produced by phage treated layer hens in groups T2 and T3 versus T1 (p<0.05) (Fig. 2a and b). At day 7, 80%, 45% and 65% eggs were positive for *Salmonella* in eggs produced by hens in groups T1, T2 and T3 respectively. By day 28, 78%, 30% and 54% of eggs were positive for *Salmonella* in eggs produced by hens in groups T1, T2 and T3 respectively (Figs. 2a and b).

### Layer bird performance and egg quality

There were no significant differences in egg production, feed intake, egg mass, body weight, egg weight, percentage of eggshell defects or egg mass across groups T1, T2 and T3 over 28 days (Table 2). There was a significant difference in feed intake in 7 days post phage treatment where layer hens in groups T2 and T3 consumed more feed than in T1 (p=0.0007). Feed intake was 109, 111 and 113 g for T1, T2 and T3 layer hens respectively (Table 2). There was also a significant difference in feed intake per egg on day 21 where intake was lower in groups T1 and T2 in comparison to T3 (p=0.017). Feed intake was 136.1, 131.4 and 147.6 g for T1, T2 and T3 layer hens respectively (Table 2).

**Table 2:** Average egg production parameters of layer hens from days 7, 14, 21 and 28.

**Day 7**									
		**Performance parameters**^1^							
**Treatment group**		**EP (%)**	**FI/layer (g)**	**FI/egg (g)**	**FI/EM (g)**	**BW (g)**	**EW (g)**	**ESD (%)**	**EM (g/layer/day)**
**T1**		77.0	109	145.8	2.5	1577.0	58.6	2.0	45.2
**T2**		80.6	111	140.9	2.4	1539.5	58.9	2.4	47.5
**T3**		81.8	113	138.7	2.3	1567.4	59.8	3.8	48.9
	LSD 5%	18	5.3	33.8	0.6	111.1	6.1	6.0	12.3
	P	0.3678	0.0007	0.5405	0.3554	0.2018	0.5660	0.2799	0.3089
	R-Square	0.0345	0.2266	0.0214	0.0356	0.0546	0.0198	0.0437	0.0404
	RMSE	10.9843	3.2257	20.6593	0.3921	67.9421	3.7431	3.6565	7.5326

**Day 14**									
**T1**		78.8	109	142.3	2.4	1577.0	58.9	1.4	46.5
**T2**		80.3	110	139.7	2.4	1539.5	58.5	2.2	47.0
**T3**		81.1	111	138.4	2.4	1567.4	58.3	1.0	47.3
	LSD 5%	18.1	4.6	33.0	0.6	111.1	3.7	4.2	11.2
	P	0.7967	0.1404	0.8244	0.9120	0.2018	0.6922	0.3453	0.9334
	R-Square	0.0079	0.0666	0.0068	0.0032	0.0546	0.0128	0.0366	0.0024
	RMSE	11.0662	2.8333	20.1638	0.3625	67.9421	2.2630	2.5539	6.8498

**Day 21**									
**T1**		80.4	110	136.1	2.3	1577.0	59.6	0.2	47.8
**T2**		80.9	111	131.4	2.3	1539.5	59.8	1.6	48.3
**T3**		81.8	111	147.6	2.4	1594.7	58.3	1.0	47.8
	LSD 5%	15.3	5.3	6.0	0.4	169.9	4.9	3.4	9.4
	P	0.8784	0.4012	0.017	0.9128	0.2382	0.2568	0.1262	0.9389
	R-Square	0.0045	0.0316	0.1332	0.0032	0.0491	0.0466	0.0700	0.0022
	RMSE	9.3522	3.6894	17.8506	0.2908	73.8927	2.9778	2.1010	5.7340

**Day 28**									
**T1**		80.5	110	140.3	2.3	1583.6	61.3	6.4	49.4
**T2**		81.1	112	132.7	2.4	1559.9	59.1	2.0	47.9
**T3**		82.1	112	141.3	2.3	1573.3	59.6	3.7	48.9
	LSD 5%	11.9	4.9	20.5	0.4	120.8	5.0	9.6	8.5
	P	0.7688	0.139	0.0678	0.4943	0.6004	0.0696	0.065	0.6576
	R-Square	0.0092	0.0669	0.0901	0.0244	0.0177	0.0893	0.0915	0.0146
	RMSE	7.3079	2.9826	12.5681	0.2236	73.905	3.0554	5.8735	5.1855

1 EP egg production percentage = amount of eggs / number of layers x 100; FI/layer (g) = average daily feed intake per layer; FI/egg (g) = average feed intake required to produce one egg; FI/EM (g) = average feed intake required to produce 1g of egg mass; BW (g) = average layer bodyweight; EW (g) = average egg weight; ESD (%) = percentage of eggs produced with eggshell defects (cracks & other abnormalities etc); EM (g/layer/day) = average amount of egg mass produced per lay per day. P probability, N number of replicates, SEM standard error mean, RMSE root mean squared error.

Furthermore, there were no statistically significant differences in egg quality parameters: egg weight, egg density, egg breaking strength, haugh unit score, shell weight and shell thickness between treatment groups (Table 3). The haugh unit scores were also not statistically significant between groups, which is a measure of egg protein quality based on the height of its egg white. Thus, phage treatment did not impact egg quality (Table 3).

**Table 3:** Egg quality variables produced by layer hens collected on days 14 and 35 (n=60).

		**Performance parameters**^1^						
**Treatment groups**		**EW (g)**	**ED (g/cm^3^)**	**ES (N)**	**HU**	**SW (g)**	**ST (mm)**	**SP, %**
**Day 14**								
**1**		59.8	1.0	19.9	77.1	5.67	0.4	9.5
**2**		59.8	1.0	19.5	74.9	5.63	0.4	9.4
**3**		58.5	1.0	18.4	75.5	5.65	0.4	9.9
	LSD 5%	9.0	0.0	8.7	16.1	0.9	0.1	3.1
	P	0.342	0.432	0.320	0.445	0.932	0.414	0.312
	SEM	0.418	0.001	0.404	0.736	0.041	0.002	0.141
	R-square	0.012	0.010	0.013	0.010	0.008	0.010	0.013
	RMSE	5.596	0.009	5.405	9.990	0.551	0.033	1.898

**Day 35**								
**1**		59.6	1.0	17.1	73.7	5.82	0.4	9.8
**2**		59.8	1.0	18.5	73.9	5.90	0.4	9.9
**3**		58.6	1.0	17.6	71.6	5.78	0.4	9.9
	LSD 5%	9.0	0.0	9.8	15.2	0.9	0.1	1.3
	P	0.437	0.412	0.418	0.392	0.494	0.790	0.643
	SEM	0.408	0.000	0.445	0.694	0.041	0.003	0.061
	R-square	0.009	0.010	0.010	0.010	0.558	0.003	0.005
	RMSE	5.559	0.006	6.063	9.445	0.558	0.043	0.825

1 EW egg weight; ED egg density, ES egg breaking strength, HU haugh unit score, SW shell weight, ST shell thickness, SP shell percent, P probability, N number of replicates, SEM standard error mean, RMSE root mean squared error.

### Phage egg spray study

**Fig. 3:**
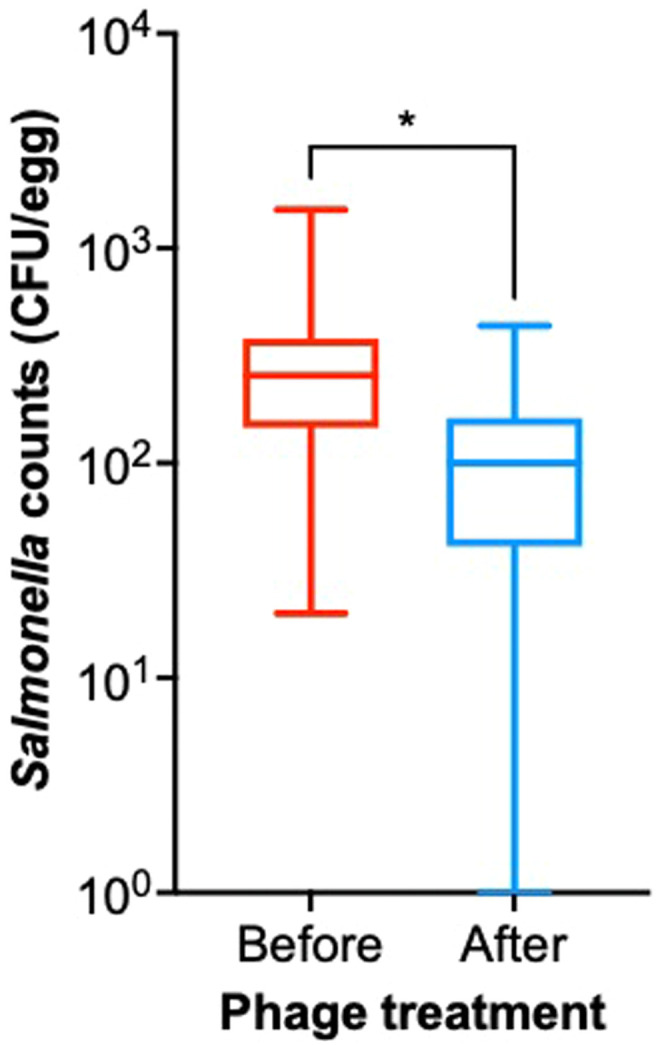
*Salmonella* abundance before and post phage treatment on *Salmonella* positive eggs (n=132). Statistical differences between groups are displayed on the graph (ns p>0.05 and * p<0.05).

A total of 391 eggs produced by layer hens in T1 were screened, of which 132 were *Salmonella* positive. These 132 eggs had average *Salmonella* abundance of 3.10×10^2^ CFU/egg and were sprayed with phage at a dose of 10^6^ PFU/litre (Fig. 3). After phage treatment, average *Salmonella* abundance was significantly (p<0.05) reduced to 1.16×10^2^ CFU/egg. Furthermore, in 13% (17/132) of eggs sprayed with the phage cocktail, the average *Salmonella* abundance was 0.00 CFU/egg (Fig. 3).

## Discussion

Poultry and their derived products are major sources of foodborne infections in humans. Although the poultry industry follows good manufacturing practices throughout the supply chain, controlling *Salmonella* in the food chain continues to be a challenge. Furthermore, it can be difficult to spot *Salmonella* infection as infected birds including layer hens often show no clinical symptoms, but contaminated eggs produced by infected hens pose a food safety risk (Gast et al., 2024). If infection in birds is detected, antibiotics are used for treatment. However, due to prevalence of antibiotic resistance alternative antimicrobials are needed to control *Salmonella* contaminating layer hens and the eggs they produce (Shaji et al., 2023). In our study we showed phage treatment offers a promising solution to controlling *Salmonella*. The phage cocktail was delivered in water for 28 days, which significantly reduced *Salmonella* shedding in layer hens and reduced its presence on the surface of eggs produced by treated layers.

In this study we tested two phage doses: 10^6^ PFU/day and 10^5^ PFU/day, selected based on data from our previous broiler bird study (Thanki et al*.,* 2023). In that broiler study we showed that the lower dose of 10^5^ PFU/day cleared infection, as shown by the observation that *Salmonella* was not recovered from faecal or caecal samples from challenged broiler hens. The dose of 10^6^ PFU/day also significantly reduced *Salmonella* abundance, thus we tested both doses (Thanki et al*.,* 2023). Our current layer hen study showed both doses consistently significantly reduced *Salmonella* faecal shedding. We also showed that *Salmonella* was reisolated from fewer cages and fewer eggs were *Salmonella* positive. However, the 10^6^ PFU/day dose was more efficacious at reducing *Salmonella* shedding and egg contamination. One potential explanation could be a higher phage dose was needed to lyse the infecting *Salmonella* strain(s).

Efficacy of phage treatment to reduce *Salmonella* colonisation and shedding in laying hens has been documented by others, who have delivered single phage or phage cocktails in feed or water. Similar to our study, Lim et al (2020) previously showed a single phage delivered at dose 10^6^ PFU/kg in feed, significantly reduced *Salmonella* colonisation in layer hens challenged with *S*. Gallinarum. Indeed, they found the challenge strain was not reisolated from the liver, spleen or caecum from infected and phage treated hens over 21 days (Lim et al*.,* 2011). In another study conducted by Zhao et al (2012) feed was supplemented with a phage cocktail at volumes of 0.020%. 0.035% and 0.050% in total feed. The concentration of the three-phage cocktail used was 10^8^ PFU/g, which was higher than our study. Like our study, birds were not challenged with *Salmonella*. They showed phage treatment reduced *Salmonella* faecal shedding from 3.47 log_10_CFU/g in *Salmonella* infected groups to ∼2.11 log_10_CFU/g in all phage treated groups over 6 weeks. They found no significant differences between the phage inclusion volumes tested, which could be due to the rate only varying by 0.015% (Zhao et al*.,* 2012). In comparison, in our trial as the dose varied by 10-fold and we observed significant differences in efficacy between the doses.

Our data shows that phage treatment delivered in water to layer hens is effective in reducing *Salmonella* shedding in layer hens and reduces *Salmonella* abundance on eggshell surfaces on their eggs. Our hypothesis is the phage cocktail may have effectively transitioned through the intestinal mucosa to reach the reproductive tract, which resulted in the observed reduction in *Salmonella* abundance on eggshell surfaces. Although we did not sample the ovaries to monitor abundance to validate this, but a recent study conducted by Villamizar et al, 2024 suggests phages could be re-isolated from ovaries of layer hens. However, it should be noted that they screened for phages in a pooled organ pool that included ovaries, bone marrow, liver and spleen samples. Therefore, it is difficult to conclude phages were present in ovaries (Hernández Villamizar et al*.,* 2024).

Overall, the performance of layer hens and egg quality were not negatively impacted by phage treatment. The performance of layer hens between the phage-treated and untreated groups was comparable. The only significant differences were at day 7 where feed intake was higher in the phage-treated groups T2 and T3 versus T1. There was also a difference in feed intake per egg on day 21, where intake was lower in groups T1 and T2 in comparison to T3. These fluctuations in feed intake could be due to several factors such as environmental and behavioural factors, which includes weather or stress. For egg quality there were no significant differences between groups, which suggests phages are safe and do not impact egg production or quality. Similar results were found by Villamizar et al, 2024 and Zhao et al, 2012, providing further evidence phages are safe to use and with no recorded side effects (Villamizar et al*.,* 2024; Zhao et al*.,* 2012).

Furthermore, the same cocktail demonstrated biocontrol potential when applied directly to eggshells, producing a marked reduction in *Salmonella abundance* within three hours. Comparable studies by Wang et al*.* (2025) and Braz et al*.* (2025), which used much higher phage doses (10⁸–10⁹ PFU/mL) against artificially inoculated eggs, achieved only modest (<2 Log₁₀ CFU/egg) reductions (Wang et al*.,* 2025; Braz et al*.,* 2025). In comparison to these studies our lower-dose, short-contact phage treatment was efficacious under naturally contaminated conditions. This underscores the potency and practical applicability of our phage cocktail for on-farm *Salmonella* control. Though direct quantitative log-reduction comparisons are difficult due to difference in methodologies used between studies.

It should be noted that most comparison studies discussed here used experimentally challenged birds and eggs to test phage efficacy. However, in our phage efficacy study we used environmentally infected layer hens and their *Salmonella* positive eggs they produced, which is more representative of on-farm infections. Also, it was unknown the serotype of the infecting *Salmonella* strain or strains and no preliminary screening was carried out to determine if our phage cocktail lysed the infection strain(s). Therefore, our study is representative of a real on-farm situation, where the infecting *Salmonella* strain(s) serotype could be unknown prior to phage treatment. Our phage cocktail was effective in this scenario at reducing *Salmonella* colonisation and shedding.

In summary, our phage cocktail delivered in drinking water at dose 10^6^ PFU/day significantly reduced *Salmonella* shedding over four weeks in environmentally infected layer hens and did not negatively impact their performance. The phage cocktail also significantly reduced *Salmonella* abundance on the eggshell surface of eggs produced by phage treated layer hens. Furthermore, the phage cocktail could be used as a disinfectant as it significantly reduced *Salmonella* abundance on eggshell when directly sprayed onto this surface. This data further supports our extensive efficacy data highlighting the effectiveness of phage therapy against *Salmonella*.

## CRediT authorship contribution statement

**Anisha M. Thanki:** Writing – original draft, Project administration, Investigation, Formal analysis. **Natasha Whenham:** Project administration, Methodology, Conceptualization. **Tom Dale:** Writing – review & editing, Formal analysis. **Mike R. Bedford:** Writing – review & editing, Formal analysis. **Helen V. Masey O’Neill:** Supervision, Project administration. **Martha R.J. Clokie:** Writing – review & editing, Supervision, Funding acquisition.

## Disclosures

The authors declare that they have no known competing financial interests or personal relationships that could have appeared to influence the work reported in this paper.
